# Inter-Specialty Controversies on the Treatment of Cardiovascular Diseases during Pregnancy: A Questionnaire Study

**DOI:** 10.3390/medicina58010138

**Published:** 2022-01-17

**Authors:** Dominika Dziadosz, Katarzyna Dudzic, Irmina Morawska, Dominika Topolska, Katarzyna Urban, Katarzyna Mizia-Stec, Łukasz J. Krzych

**Affiliations:** 1 Upper Silesian Medical Centre, Students’ Scientific Society of the First Department of Cardiology, School of Medicine in Katowice, Medical University of Silesia, 40-055 Katowice, Poland; dominika.dziadosz@gmail.com (D.D.); irmina.morawska@gmail.com (I.M.); 2 Students’ Scientific Society, Department of Anesthesiology and Intensive Care, School of Medicine in Katowice, Medical University of Silesia, 40-752 Katowice, Poland; dudzic.kat@gmail.com (K.D.); dotciat@wp.pl (D.T.); kasiaurban@gmail.com (K.U.); 3 First Department of Cardiology, School of Medicine in Katowice, Medical University of Silesia, 40-055 Katowice, Poland; 4 Department of Anaesthesiology and Intensive Care, Faculty of Medical Sciences in Katowice, Medical University of Silesia, 40-752 Katowice, Poland; l.krzych@wp.pl

**Keywords:** pregnancy, cardiovascular disease, pulmonary hypertension, pulmonary embolism, heart failure

## Abstract

*Background and Objectives*: Management of cardiovascular disease (CVD) during pregnancy is challenging and usually requires eminence-based decisions due to limited strong-evidence data in this field. The purpose of our study was to compare the attitudes of anaesthesiologists, cardiologists, and gynaecologists towards the diagnosis and treatment of potentially life-threatening CVDs during pregnancy. *Materials and Methods*: A cross-sectional, questionnaire-based study was performed among 111 doctors (55 anaesthesiologists, 36 cardiologists, 20 gynaecologists). Personal opinions on the recommendations (*n* = 19) regarding rare, potentially life-threatening CVDs during pregnancy were recorded using a five-item Likert scale. *Results*: Opinions regarding eight statements (42%) varied substantially between specialties (*p* < 0.05). The most distinctive differences between physicians concerned the following recommendations: “thrombolysis should only be used in pulmonary embolism with cardiogenic shock” (agree: 52.7% of anaesthesiologists, 80.4% of cardiologists, 25.0% of gynaecologists; *p* < 0.001); “women with the antiphospholipid syndrome should restart treatment with vitamin K antagonists from the second trimester of pregnancy” (agree: 12.7% of anaesthesiologists, 69.4% of cardiologists, 20.0% of gynaecologists; *p* < 0.001); “women with symptomatic pulmonary hypertension should have a Swan–Ganz catheter inserted for labour” (agree: 20.0% of anaesthesiologists, 11.1% of cardiologists, 55.0% of gynaecologists; *p* = 0.001). *Conclusions*: Physicians’ opinions regarding diagnostics and treatment of CVDs in pregnancy remain controversial. A multidisciplinary approach is recommended to ensure the safety and effectiveness of management in these unique medical conditions.

## 1. Introduction

Pregnancy is a physiologic dynamic process associated with significant and possibly reversible hemodynamic changes in the cardiovascular system, which provide for the proper growth and development of the foetus and prepare the mother’s body for delivery and the postpartum period. However, adaptive changes such as increased cardiac output, arterial compliance and the extracellular fluid volume, and lowered blood pressure and total vascular resistance may exacerbate symptoms of existing CVD and substantially increase the risk of maternal and foetal death [1].

Thanks to developments in diagnostics and therapy, young women’s survival with congenital heart defects has substantially increased. Currently, over 90% of them live to adulthood [2]. Some of them refuse to use contraception and attempt to deliver their baby safely. It is estimated that 1–4% of women will experience pregnancy complicated by cardiovascular diseases (CVD), accounting for up to 15% of maternal mortality [3]. Due to the growing population of pregnant women suffering from CVD, in 2018, the European Society of Cardiology (ESC) published detailed guidelines for treating those patients [4], putting the most significant impact on multidisciplinary cooperation between gynaecologists, cardiologists, and anaesthesiologists.

The estimation of maternal cardiovascular risk should be followed by a modified World Health Organization classification (mWHO), which is divided into classes I–IV, in the proper sequence from no detectable (I) to very high risk (IV). Pulmonary arterial hypertension or severe symptomatic aortic stenosis are assessed as mWHO class IV and have a particularly high risk of maternal mortality. In those cases, according to mWHO, pregnancy is contraindicated, and if pregnancy occurs, its termination should be taken into consideration [4]. On the other hand, clinical “grey area” situations require careful risk stratification to make the best decision for both patients—the mother and the child. Due to the lack of specific guidelines, effective cooperation between medical specialities is necessary to ensure the best individual management of those patients.

Our study aimed to compare the attitudes of anaesthesiologists, cardiologists, and gynaecologists towards the diagnosis and treatment of potentially life-threatening CVDs during pregnancy.

## 2. Materials and Methods

The cross-sectional study was conducted from December 2019 to May 2020 in 14 Polish tertiary medical centres with cardiology, anaesthesiology and intensive care, and gynaecology and obstetrics departments with experience in the treatment of CVDs in pregnancy. Regarding the participation in the study, inclusion criteria were as follows: specialisation or ongoing formal training (residency) in the fields of cardiology, anaesthesiology, and gynaecology and obstetrics.

An anonymous self-prepared questionnaire (Appendix A) was distributed among Polish medical professionals via email as an online questionnaire. The questionnaire consisted of 19 recommendations based on the ESC guidelines for the treatment of CVDs in pregnancy [4] and for the treatment of adult congenital heart diseases [5] and was divided into four categories: “Pharmacotherapy”, “States of instant danger to life”, “Procedures”, and “Delivery and Postpartum”.

The survey was designed by co-authors of the study specialized in the field of cardiology as well as anaesthesiology and intensive care; however, it was not externally validated. The questionnaire was used exclusively by the authors during the research.

Out of 300 invited physicians, 111 (48 males, 38 ± 11 years of age) responded to the questionnaire (response rate: 37%). The study included 55 anaesthesiologists, 36 cardiologists, and 20 gynaecologists—57 specialists and 54 residents. Respondents gave their personal opinions using the 5-point Likert Scale (1—I strongly disagree, 2—I disagree, 3—I’m neutral, 4—I agree, and 5—I strongly agree). Points “4” and “5” were combined into one group and treated equally as a positive opinion, while points “1” and “2” were treated as a negative opinion.

The bioethics committee waived the requirement for written consent to conduct the study (No. PCN/0022/KB/291/I/19/20).

The statistical analysis was performed using Statistica 12.0 software (StatSoft, Cracow, Poland). Qualitative data was presented as crude value and percent. The chi-square test was used to verify between-group differences. A *p*-value < 0.05 was considered statistically significant.

## 3. Results

Statistically significant (*p* < 0.05) between-group differences were observed in 8 out of 19 recommendations. The most important difference was recorded in the “Pharmacotherapy” category.

**Figure 1:**
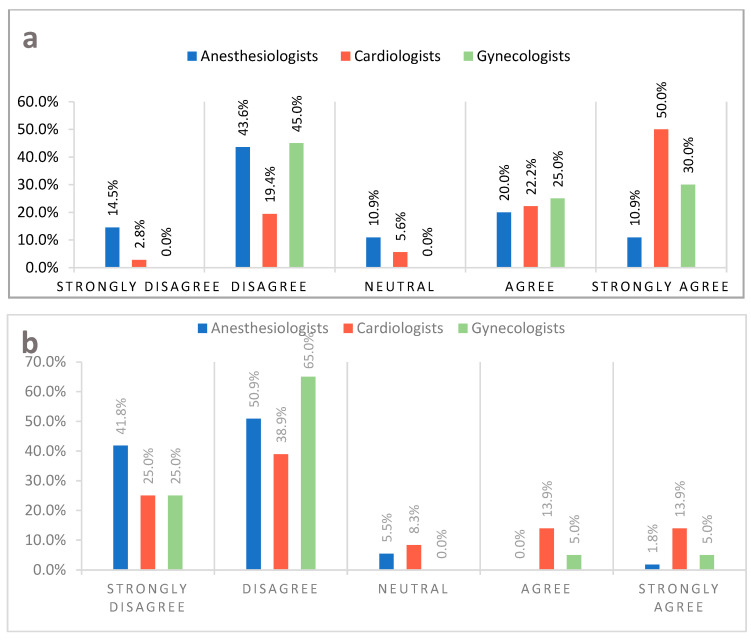
(**a**) Every woman with idiopathic pulmonary hypertension in reproductive age, regardless of the severity of the disease, should use an effective method of contraception. (**b**) Women with heart failure and a reduced ejection fraction, regardless of the puerperal clinical condition, should be discouraged from breastfeeding.

The use of contraception by women suffering from iPAH was the main point of contention between doctors (Figure 1a). Remaining questions in this category regarded: the treatment of idiopathic pulmonary hypertension (iPAH), and antiphospholipid syndrome (APS), and the use of volatile anaesthetics and β-blockers during pregnancy (Table 1).

The section “Delivery and Postpartum” delivered controversial answers as well. Breastfeeding by women with heart failure and a reduced ejection fraction (Figure 1b), insertion of a Swan–Ganz catheter for the delivery, and planned coronary angioplasty in the postpartum period garnered the most significant difference in responses of the surveyed physicians (Table 1).

The “States of Instant Danger to Life” section was the third most contentious category. The question with the most varied responses regarded thrombolysis in pregnant patients suffering from pulmonary embolism (PE) (Table 1).

Responses of specialists and residents were statistically significant only in one question, which regarded valvular disease which occurs rarely in pregnant patients. Both groups had consistent opinions on the rest of the clinical problems presented in the questionnaire.

Answers of all surveyed physicians are illustrated in the Table 2.

## 4. Discussion

Our study aimed to compare the opinions of anaesthesiologists, gynaecologists, and cardiologists on the methods of diagnosis and treatment of cardiological diseases that potentially threaten the life and health of both mother and child. The results of our survey showed diverse opinions in many aspects.

### 4.1. Pharmacotherapy

Idiopathic pulmonary arterial hypertension (iPAH)—defined as pulmonary artery pressure (PAP) greater than 20 mmHg at rest and elevated pulmonary vascular resistance (PVR) of more than 3 Wood units assessed during catheterisation of the right heart—is a rare disease associated with a poor outcome among pregnant patients, with a mortality rate of 30–56%, and the highest risk of maternal death during birth and the postpartum period. Furthermore, iPAH has a high foetal and newborn mortality rate at 30% [4]. This debilitating condition is more common in women and mainly affects women of childbearing age [6]. Moreover, it can present its first symptoms during pregnancy [4].

According to the ESC guidelines [4], women suffering from iPAH should avoid pregnancy, which accentuates the importance of an individual approach to the patient and the necessity of thorough counselling. It is crucial to advise women burdened by iPAH to use an effective method of contraception with progestin-only contraceptives, which are the safest choice in this group of patients because estrogenic contraceptives elevate the risk of venous thromboembolism (VTE) and have a damaging effect on the already-strained pulmonary vasculature. Progestin-releasing or copper intrauterine devices (IUDs) can be considered, but it is important to note that dilation of the cervix during IUD implantation can cause a vasovagal response, which can be very dangerous to patients with iPAH [6].

When pregnancy occurs and the patient decides to carry on with the pregnancy, despite appropriate counselling on potential risks of gestation, it is recommended to refer the pregnant patient to a multidisciplinary specialist centre with ensured complex care by pulmonary hypertension specialists, obstetricians, neonatologists, and critical care physicians, to provide the highest standard of treatment, with a detailed delivery time and mode [7]. Guidelines recommend considering planned early hospitalisation, because those patients should not go into labour naturally, to reduce the risk of birth at night or on the weekend with the assistance of an inexperienced team. When it comes to a detailed birth plan, the decision on vaginal or caesarean delivery has to be made individually for each patient, considering the potential risks and benefits of both methods. Careful post-natal care plays a vital role in the successful management of those patients, because most deaths occur at this stage [8].

In light of current knowledge, the results of our study stand in line with current recommendations—48.6% of surveyed physicians, mainly 72.2% cardiologists, agreed that women suffering from iPAH should be advised against pregnancy and familiarised with effective contraception methods. It is noteworthy that 58.1% of surveyed anaesthesiologists disagreed with the guideline. In our opinion, the attitude of anaesthesiologists regarding this topic requires further research.

β-blockers are one of the most prescribed classes of drugs in cardiology [9]. They are widely used to treat several cardiovascular conditions, such as tachycardia, arterial hypertension (HA), myocardial infarction (MI), congestive heart failure (CHF), arrhythmias, and many more [10]. All of the previously mentioned diseases can occur during pregnancy. Although very beneficial, the use of β-blockers can lead to many adverse effects, especially in pregnant patients. The blockade of β-receptors causes the constriction of placental vessels and results in decreased uteroplacental blood flow. Disturbance in placental hemodynamic results in foetal hypoxemia, IUGR, as well as increased risk of infants being born pre-term and small for gestational age (SGA) [10,11,12,13]. Even though these adverse effects are acknowledged, sometimes the potential benefits of the use of β-blockers outweigh the risks. For example, “Control of Hypertension in Pregnancy Study” [4] has shown that strict management of hypertension results in the reduction of adverse outcomes, such as preeclampsia [4].

Though labetalol is a drug of choice in the treatment of HA during pregnancy [4], there are published studies reporting the correlation between labetalol use and significantly increased risk of IUGR [14]. This discrepancy has been reported in our research as well, with 53.1% of respondents considering almost all β-blockers to be risk factors for intrauterine developmental delay, and a little less, 41.4% not agreeing with this statement.

Another disproportion of opinions arose on the treatment of APS in pregnant women—54.9% of doctors were against anticoagulant therapy using vitamin K antagonists (VKA) during pregnancy. However, 32.4% of doctors agreed that VKAs could be administered from the second trimester of pregnancy. Research conducted by Rai and colleagues [15] revealed that 90% of women with APS who declined anticoagulant therapy miscarried during the first trimester of pregnancy. VKAs are the drugs of choice for secondary prevention after a thrombotic event in patients with APS [16]; however, they are not recommended during pregnancy [4]. Despite that, VKAs seem relatively safe to administer 2–4 weeks before childbirth. While it is possible, it still carries the risk of foetal complications such as intracranial bleeding and central nervous system defects; while this complex topic remains highly controversial, it is advisable to minimise the risk of thrombotic complications by using fractionated and unfractionated heparin [17].

It is estimated that 2% of women undergo non-obstetric surgery during pregnancy mainly due to conditions, such as trauma, appendicitis, or ovarian disorders [18]. The mortality of pregnant women undergoing surgery is not significantly higher than that of women who are not pregnant [19]. However, providing anaesthesia for pregnant women, especially those burdened by CVD, is more challenging. It requires seeking the best outcome for the mother in the safest possible way to protect her child. To provide the best standard of care, the anaesthesiologists must avoid potentially dangerous drugs that can affect the foetus, ensure sufficient uteroplacental flow, and inhibit uterine contractions leading to pre-term delivery while maintaining adequate anaesthesia [20]. It is important to mention that almost every drug can be potentially teratogenic if given in a high enough dose [21]. Published studies show a correlation between volatile anaesthetics and damage to the developing animal central nervous system [22]. For example, sevoflurane can inhibit neuronal proliferation and promote neurophotonic among rat offspring. However, we cannot apply this data to humans because of interspecies variation and higher doses of drugs used in those studies. Many extensive retrospective studies failed to show teratogenic effects of anaesthetic agents used during surgery in the first trimester of pregnancy [23,24,25,26,27,28,29]. Taking it all into consideration, it is hardly surprising that over 63.7% of surveyed anaesthesiologists did not consent to the necessity to minimise the use of inhalation anaesthetics.

Current studies show that administration of nitrous oxide and volatile, opioid, regional, and local anaesthesia have no significant effect on the foetus. Furthermore, there is a lack of evidence of adverse neonatal outcomes. Patients’ primary disease or surgery has a higher risk of causing abortion than exposure to anaesthetic agents [30].

It is estimated that 2% of women undergo non-obstetric surgery during pregnancy mainly due to conditions such as trauma, appendicitis, or ovarian disorders [18]. The mortality of pregnant women undergoing surgery is not significantly higher than that of women who are not pregnant [19]. However, providing anaesthesia for pregnant women burdened by CVD is significantly more challenging. It is important to mention that almost every drug can be potentially teratogenic if given in a high enough dose [20]. Published studies show a correlation between volatile anaesthetics and damage to the developing animal central nervous system [21]. However, we cannot apply this data to humans because of interspecies variation and higher doses of drugs used in those studies. Current studies show that administration of nitrous oxide, volatile, opioid, regional, and local anaesthesia have no significant effect on the foetus [22,23,24,25,26,27,28]. Patients’ primary disease or surgery has a higher risk of causing abortion than exposure to anaesthetic agents [29]. Taking it all into consideration, it is hardly surprising that over 63.7% of surveyed anaesthesiologists did not consent to the necessity to minimise the use of inhalation anaesthetics.

### 4.2. States of Instant Danger to Life

Pulmonary embolism (PE) is one of the leading causes of death in pregnant women in developed countries. The mortality rate due to PE is at 1.1–1.5/100,000 births in the US, Europe, and the UK. The risk of venous thromboembolism (VTE), including PE, is more than five times higher in pregnant women than in non-pregnant women [30]. Guidelines indicate thrombolysis as the treatment of choice for pulmonary embolism for non-pregnant patients [31]. We asked doctors if they agreed that thrombolysis should be used only in patients with massive pulmonary embolism complicated by cardiogenic shock. It turned out that 80.4% of cardiologists, 52.7% of anaesthesiologists, and 25% of gynaecologists agreed with the above statement. It is worth noting that 70% of gynaecologists disagreed with the above claim. Due to insufficient clinical data on the use of thrombolysis in pregnant women, the European Society of Cardiology guidelines recommends using thrombolytic drugs only when pulmonary embolism coexists with severe hypotension and shock [4]. So far, the literature reports 200 cases of patients who were treated with streptokinase and then recombinant tissue plasminogen activator, alteplase. Six percent of these women had a foetal loss and premature delivery. Moreover, it is worth adding that the latest data describes 23 cases of pregnant women with massive pulmonary embolism who were treated with systemic thrombolysis [32]. The use of thrombolysis as a method of pulmonary embolism treatment in pregnant women is associated with an increased risk of bleeding complications and foetal death. However, randomised trials have proven that thrombolytic therapy is more effective than heparin in reducing clot burden and improving patients’ hemodynamic status. There is lack of data available comparing the long-term survival of patients treated with thrombolytic agents vs. heparin. Thus, no agent has been proven to be more effective than others [33].

### 4.3. Procedures

The questions regarding invasive interventions in pregnant women resulted in high consensus among specialists and stayed in line with recommendations [4].

### 4.4. Delivery and Postpartum

In our research, 92.7% of anaesthesiologists, 63.9% of cardiologists, and 90% of gynaecologists stated that breastfeeding should be encouraged amongst women with HF and a reduced ejection fraction, irrespectively of the clinical state of the mother during the peripartum period. There is a lack of data regarding breastfeeding by critically ill women, including those suffering from HF [34]. The decision on continuation or cessation of breastfeeding amongst women with HF, NYHA class one and two, should be made after thoroughly considering potential risks and benefits. Interestingly, the results of our study differ significantly from current ESC guidelines. Women with HF, a reduced ejection fraction (HFrEF), and NYHA classes 3 and 4 ought to be discouraged from breastfeeding [4]. Discontinuation of breastfeeding reduces metabolic demand and allows early optimal treatment of HF.

### 4.5. Limitations of the Study

This study has a few limitations. The research covered only Polish medical centres; moreover, it contained a limited number of participants, which is illustrated by the low questionnaire response rate (37%). The questionnaire was created specifically for the study and it was not used in other studies. It was not externally validated. However, it was thoroughly examined by the co-authors of the study, who specialize in the field of anaesthesiology and intensive care and cardiology.

During our research, we came across several confounding factors. Even though the well-being of mother and the child should be the primary therapeutic goal of treatment, different specialists can have varying or even opposing priorities due to their field of expertise. Moreover, methods of achieving the best result for the patient can vary, resulting in bias caused by individual eminence-based decisions due to diverse clinical experience. The forementioned factors can contribute to contrary and subjective decisions while completing the survey.

Furthermore, recommendations on CVD treatment during pregnancy are often not the result of randomised controlled trials but the effect of specialists’ long-term experiences, clinical case reports, and small study groups.

## 5. Conclusions

Response variability of cardiologists, anaesthesiologists, and gynaecologists confirms that multidisciplinary decisions are necessary to provide the best standard of care for pregnant women with CVDs, especially when there are no clear guidelines regarding diagnostics and treatment. The uneven number of responses may suggest that there might not be enough specialists with appropriate clinical experience to form multidisciplinary teams able to provide a holistic and individual treatment plan for these challenging patients.

We believe that further research in this field as well as multidisciplinary care and use of the newest clinical knowledge play a key role in improving patients’ care and outcomes. A patient-centred approach is a cornerstone for ensuring the highest standard of care in these complex medical entities.

## Figures and Tables

**Table 1:** Inter-specialty response variability among surveyed physicians (A—anaesthesiologists, C—cardiologists, G—gynaecologists).

	Strongly Disagree			Disagree			Neutral			Agree			Strongly Agree		
**PHARMACOTHERAPY**															
1.	Pregnant women with antiphospholipid syndrome (APS) are recommended to restart treatment with vitamin K antagonists from the second trimester of pregnancy.														
	A	C	G	A	C	G	A	C	G	A	C	G	A	C	G
%	20.0	19.4	20.0	43.6	8.3	60.0	23.6	2.8	0.0	65.5	50.0	20.0	9.1	19.4	0.0
2.	Inhalation anaesthetics should be avoided in pregnant women undergoing non-obstetric surgeries during pregnancy.														
	A	C	G	A	C	G	A	C	G	A	C	G	A	C	G
%	25.5	11.1	5.0	38.2	22.2	35.5	7.2	33.3	5.0	20.0	27.8	45.0	9.1	5.6	10.0
3.	The use of almost all β-blockers in pregnancy may be associated with a risk of intrauterine foetal development delay.														
	A	C	G	A	C	G	A	C	G	A	C	G	A	C	G
%	5.5	0.0	10.0	32.7	33.3	55.0	7.3	5.6	0.0	52.7	38.9	15.0	1.8	22.2	20.0
**DELIVERY AND POSTPARTUM**															
4.	Every pregnant woman with symptomatic pulmonary hypertension should have a Swan–Ganz catheter inserted for labour.														
	A	C	G	A	C	G	A	C	G	A	C	G	A	C	G
%	16.4	33.3	0.0	52.7	38.9	15.0	10.9	16.7	30.0	14.5	8.3	40.0	5.5	2.8	15.0
5.	If it is clinically indicated, planned coronary angioplasty should be performed in pregnant women after the delivery.														
	A	C	G	A	C	G	A	C	G	A	C	G	A	C	G
%	7.3	0.0	0.0	10.9	19.4	0.0	10.9	5.6	10.0	65.5	47.2	65.0	5.5	27.8	25.0
**STATES OF INSTANT DANGER TO LIFE**															
6.	Within pregnant women with pulmonary embolism, thrombolytic therapy should be used only in the event of cardiogenic shock.														
	A	C	G	A	C	G	A	C	G	A	C	G	A	C	G
%	7.3	11.1	35.0	34.5	5.6	35.0	5.5	2.8	5.0	43.6	38.9	25.0	9.1	41.7	0.0

**Table 2:** Responses of the surveyed physicians.

Recommendation	Response (*n*) %					*p*
	Strongly Disagree	Disagree	Neutral	Agree	Strongly Agree	
**PHARMACOTHERAPHY**						
**1**	(9) 8.1%	(40) 36.0%	(8) 7.2%	(24) 21.6%	(30) 27.0%	0.002
**2**	(5) 4.5%	(41) 36.9%	(6) 5.4%	(46) 41.4%	(13) 11.7%	0.007
**3**	(22) 19.8%	(39) 35.1%	(14) 12.6%	(27) 24.3%	(9) 8.1%	<0.001
**4**	(12) 10.8%	(36) 32.4%	(2) 1.8%	(47) 42.3%	(14) 12.6%	0.210
**5**	(14) 12.6%	(42) 37.8%	(17) 15.3%	(30) 27.0%	(8) 7.2%	0.185
**6**	(19) 17.1%	(36) 32.4%	(17) 15.3%	(30) 27.0%	(9) 8.1%	0.006
**DELIVERY AND POSTPARTUM**						
**1**	(4) 3.6%	(13) 11.7%	(10) 9.0%	(66) 59.5%	(18) 16.2%	0.019
**2**	(1) 0.9%	(26) 23.4%	(7) 6.3%	(26) 23.4%	(6) 5.4%	0.353
**3**	(21) 18.9%	(46) 41.4%	(18) 16.2%	(19) 17.1%	(7) 6.3%	<0.001
**4**	(37) 33.3%	(55) 49.5%	(6) 5.4%	(6) 5.4%	(7) 6.3%	0.016
**STATES OF INSTANT DANGER TO LIFE**						
**1**	(15) 13.5%	(28) 25.2%	(5) 4.5%	(43) 38.7%	(20) 18.0%	<0.001
**2**	(14) 12.6%	(60) 54.1%	(12) 10.8%	(18) 16.2%	(7) 6.3%	0.439
**3**	(10) 9.0%	(44) 39.6%	(17) 15.3%	(33) 29.7%	(7) 6.3%	0.140
**PROCEDURES**						
**1**	(13) 11.7%	(38) 34.2%	(5) 4.5%	(37) 33.3%	(18) 16.2%	0.110
**2**	(13) 11.7%	(42) 37.8%	(17) 15.3%	(34) 30.6%	(5) 4.5%	0.078
**3**	(6) 5.4%	(11) 9.9%	(6) 5.4%	(50) 45.0%	(38) 34.2%	0.198
**4**	(0) 0.0%	(16) 14.4%	(16) 14.4%	(56) 50.5%	(23) 20.7%	0.749
**5**	(9) 8.1%	(22) 19.8%	(6) 5.4%	(44) 39.6%	(30) 27.0%	0.232
**6**	(9) 8.1%	(21) 18.9%	(4) 3.6%	(54) 48.6%	(23) 20.7%	0.394

## Data Availability

The data presented in this study are available on request from the corresponding author. The data are not publicly available due to confidentiality of the research.

## Appendix A

	**Questions**
	**PHARMACOTHERAPY**
**1**	Every woman with idiopathic pulmonary hypertension in reproductive age, regardless of the severity of the disease, should use an effective method of contraception.
**2**	The use of almost all β-blockers in pregnancy may be associated with a risk of intrauterine fetal development delay.
**3**	Pregnant women with antiphospholipid syndrome (APS) are recommended to restart treatment with vitamin K antagonists from the second trimester of pregnancy.
**4**	Routine antibiotic prophylaxis should be implemented in pregnant women with the diagnosed valvular disease who are at risk of infective endocarditis.
**5**	Within pregnant women with severe arterial hypertension, hydralazine should be used only when other pharmacological treatments are ineffective.
**6**	Inhalation anesthetics should be avoided in pregnant women undergoing non-obstetric surgeries during pregnancy.
**DELIVERY AND POSTPARTUM**	
**1**	If it is clinically indicated, planned coronary angioplasty should be performed in pregnant women after the delivery
**2**	Vaginal delivery with epidural anesthesia should be the first choice method for women with symptoms of aortic stenosis.
**3**	Every pregnant woman with symptomatic pulmonary hypertension should have a Swan–Ganz catheter inserted for labor.
**4**	Women with heart failure and a reduced ejection fraction, regardless of the puerperal clinical condition, should be discouraged from breastfeeding.
**STATES OF INSTANT DANGER TO LIFE**	
**1**	Within pregnant women with pulmonary embolism, thrombolytic therapy should be used only in the event of cardiogenic shock.
**2**	Every pregnant woman with the symptomatic valvular disease should be hospitalized in the intensive care unit, even after an uncomplicated delivery.
**3**	Each type of anesthesia increases the likelihood of developing aortocaval compression syndrome.
**PROCEDURES**	
**1**	Within women with symptoms of circulatory failure and idiopathic pulmonary hypertension, pregnancy should be terminated at its earliest stage.
**2**	A pregnant woman with diagnosed idiopathic pulmonary hypertension should undergo catheterization of the right heart in the first trimester of pregnancy.
**3**	Cesarean section should be the first-choice method for a pregnant woman with idiopathic pulmonary hypertension.
**4**	NT-proBNP levels should be measured routinely in all pregnant women with symptoms of heart failure in each trimester of pregnancy.
**5**	Within women being in reproductive age with severe aortic stenosis, percutaneous balloon valvuloplasty should be performed preventively before a planned pregnancy.
**6**	Electrical cardioversion is a safe procedure for the mother and the fetus in each trimester of pregnancy
